# Longitudinal analysis of raccoon rabies in West Virginia, 2000–2015: a preliminary investigation

**DOI:** 10.7717/peerj.4574

**Published:** 2018-04-04

**Authors:** K. Bert Plants, Sijin Wen, Jeffrey Wimsatt, Sarah Knox

**Affiliations:** 1 School of Public Health, West Virginia University, Morgantown, WV, United States of America; 2 Morgantown, WV, United States of America

**Keywords:** West Virginia, Longitudinal analysis, Rabies, Infectious disease

## Abstract

Animal borne rabies virus is a source of infection in humans, and raccoons (*Procyon lotor)* are the primary terrestrial reservoir in West Virginia (WV). To assess the behavior and status of raccoon variant rabies virus (RRV) cases in WV, a longitudinal analysis for the period 2000–2015 was performed, using data provided by the state Bureau of Public Health. The analytic approach used was negative binomial regression, with exclusion of those counties that had not experienced RRV cases in the study period, and with further examination of those counties where oral rabies vaccine (ORV) baits had been distributed as compared with non-ORV counties. These analyses indicated that there had been a reduction in numbers of RRV positive animals over the study period, predominantly due to a decrease in raccoon infections. Non-raccoon hosts did not appear to have a similar decline, however. The rates of decline for the ORV zone were found to be significantly greater as compared to the non-ORV area. The study was limited by the lack of data for season or point location of animal collection, and by lack of surveillance effort data. Even so, this study has implications for the preventive measures currently being implemented, including expanded vaccination effort in domestic animals. Spatial analyses of RRV and further examination of the virus in non-raccoon hosts are warranted.

## Introduction

Rabies lyssavirus causes an almost invariably fatal infection in any mammal, including humans. Rabies virus induced fatal encephalomyelitis is endemic throughout the Americas, with higher numbers of human deaths in Mexico, Central America and South America (Hampson et al., 2015).

Over the past 100 years, the host distribution of rabies virus and risk of human exposure in the United States have changed dramatically because of coordinated vaccination efforts in domestic animals, especially dogs and cats (Hampson et al., 2015). More than 90% of all animal cases reported annually to the CDC now occur in wildlife; whereas before 1960, the majority were in domestic animals (Centers for Disease Control and Prevention, 2011). Although rabies virus kills thousands of people each year worldwide, human deaths have decreased dramatically in the United States, primarily due to successful preventive measures (Hampson et al., 2015; Lozano et al., 2012).

Infected wildlife animals, including raccoons (*Procyon lotor*), often lose their fear of humans and become active during daylight hours, dramatically increasing the potential for human and domestic animal exposures (Kappus et al., 1970). When a dog or cat is reported to have bitten a human, the animal is quarantined for an established observation period of 10 days ( West Virginia Legislature, 2017). In the event of a wild animal bite, if the animal in question is deceased, or the quarantine period is not established, the brain of the animal is submitted for rabies virus confirmation, if available (Brown et al., 2016). Often, the person bitten must undergo a post exposure prophylactic (PEP) regimen that entails infusion of anti-rabies immunoglobulin into the wound, in addition to four doses of human rabies vaccine (Rupprecht et al., 2010). In the US, 11,000 to 36,000 PEP treatments are given to people annually, suggesting rabies virus exposure remains a significant problem (Christian et al., 2009). In the event of a local outbreak, or when the disease becomes established in a region, the number of PEP treatments administered increases to meet the local need, putting a burden on local resources (Gordon et al., 2005). While costs vary, a course of rabies immunoglobulin and four doses of vaccine typically exceed $3,000 (Meltzer & Rupprecht, 1998; Shwiff et al., 2007).

There are a number of variants of rabies virus, with many being associated with a specific host, although spillover into other hosts frequently occurs (Wallace et al., 2014). In fact, the labelling of the variants by host (raccoon, bat, fox, etc.) is only reflective of the host that acts as the primary reservoir for that variant of virus at that time (Baer, 1991). In the United States, effective rabies virus vaccination protocols for domestic animals have resulted in near elimination of the disease in that population (Rupprecht, Hanlon & Hemachudha, 2002). Nationally, the cost of rabies prophylaxis, treatment and control programs, including domestic animals and wildlife, is estimated to be between $250 and $500 million dollars annually (Meltzer & Rupprecht, 1998). Additionally, rabies virus infection in agricultural settings can be costly for animal producers (Chipman et al., 2013).

In West Virginia (WV), the viral variants known to be present are the bat and raccoon rabies virus variants (West Virginia Department of Health and Human Services, 2017b). Bat variant rabies virus cases have been reported from all counties. Due to the number of potential bat variants of rabies virus, there are probably multiple bat variants in WV (Streicker et al., 2010). Even so, bat cases represent approximately 5% of the animals found to be positive for rabies virus (West Virginia Department of Health and Human Services, 2017a). In contrast, raccoon variant rabies virus (RRV) is historically enzootic in the Southeast United States, and has expanded its range in the eastern part of WV subsequent to the inadvertent introduction of translocated rabid raccoons along the WV—Virginia border near Greenbrier County in the late 1970s (Nettles et al., 1979). Since then, RRV infection has expanded geographically to include the entire eastern seaboard, and has become enzootic throughout that region (Slate et al., 2009). This has become a major issue because, despite the success of immunization programs in domestic animals, rabies virus infection has become a persistent problem in WV wildlife, particularly in raccoons (West Virginia Department of Health and Human Services, 2017a).

Beginning in 2001 the Animal and Plant Health Inspection Service of the United States Dept. of Agriculture began an oral rabies vaccination (ORV) program in WV (Slate et al., 2002). The baits are broadcast, primarily from aircraft, in a band passing through the center of the state in a north-south orientation. The baited area was intended to act as a *cordon sanitaire* to prevent westward encroachment of RRV.

Prior to the introduction of RRV in WV, there were a handful (5–10) of rabies virus positive raccoons reported annually, all of which were infected with bat variant viruses (West Virginia Department of Health and Human Services, 2017a). Once the emergent RRV front moved through, numbers of RRV positive raccoons increased dramatically, peaking in 2002 with 126 positive animals identified (West Virginia Department of Health and Human Services, 2017a). Moreover, spillover hosts increased, adding 37 positive animals (23% of the total positives) that year, including eight domestic animals (cats, horses and cattle) (West Virginia Department of Health and Human Services, 2017a). It has been speculated that RRV may be especially associated with spillover into other hosts, and could be described as a “super spreader” organism (Wallace et al., 2014). This could indicate that RRV has the potential to undergo host shifts more readily than other viruses (Wallace et al., 2014). This ability might even provide the opportunity to create a viral reservoir in vector hosts. The importance of RRV in WV is highlighted by the fact that over 93% of rabies virus positive animals identified from 2000–2015 were infected with RRV (West Virginia Department of Health and Human Services, 2017a).

Several factors have contributed to the current situation, where over 2,500 animal bites and other potential rabies virus exposures are reported annually in WV (West Virginia Department of Health and Human Services, 2017b). Of particular interest are cats, which represent roughly 5% of RRV positives identified in peridomestic settings in WV (West Virginia Department of Health and Human Services, 2017a). This is of concern because many domestic animals, especially cats and livestock, remain unvaccinated for rabies virus, even though rabies virus prophylaxis is mandatory for dogs and cats in WV ( West Virginia Legislature, 2017). In addition, cats have a propensity to establish viable feral populations (Rupprecht, Hanlon & Slate, 2006).

In order to assess the public health risk of rabies virus to humans in WV and the current state of rabies virus prevention efforts and evaluate the temporal effects of the ORV program, the aim of this study is to determine whether there has been a significant change in the number of RRV cases over the period 2000–2015 in WV. This was accomplished by using counties where RRV was reported, and among these counties, comparing those where the ORV program has been active with those where RRV is enzootic but ORV has not been deployed. Hypotheses tested will be that there are significant reductions in RRV cases overall, and that there are significant differences between the rates of decline in the counties where ORV has been implemented as compared to the RRV enzootic counties, in all animal groups. The analytic approaches employed were used to evaluate the data while accounting for the uneven distribution of rabid animals, and while including all types of affected animal hosts in the analysis.

## Materials and Methods

### Data collection and database structure

Data used here came from the WV State Bureau of Public Health and consisted of the annual state reported rabies virus case database for RRV. These data, as used here, represented county level data for the years 2000 to 2015 (West Virginia Department of Health and Human Services, 2017a). This is a complete dataset, including all cases of rabies virus hosts by species identified in the state during the study period, the county where they were collected and viral variant.

Identifying suspect animals for the analysis used here typically relied on one of the following situations: (1) animals involved with biting or scratching humans, (2) animals involved with biting or scratching domestic animals or livestock, (3) the opportunity to observe an animal exhibiting an atypical behavior, or (4) results of an occasional environmental “spot check” of areas (Office of Laboratory Services, 2018). Also included in the database are animals collected by the US Department of Agriculture, Animal and Plant Health Inspection Service subsequent to implementation of the ORV program. Unfortunately, data are not available regarding how many of these were human exposures (defined as a bite or a scratch).

Generally, suspect animal brains were submitted by veterinarians or animal control personnel to the state diagnostic laboratory for direct fluorescent antibody screening (Office of Laboratory Services, 2018). Also, animals that tested positive subsequent to trapping and testing by the USDA Animal and Plant Health Inspection Service are included in the database. All positive samples from either source were submitted to the CDC for confirmatory testing and viral variant identification. Viral detection was performed at CDC, using direct fluorescent antibody testing, with subsequent variant typing performed using a validated RT-PCR method (McQuiston et al., 2001).

There were 1,569 animals found to be positive for any rabies virus during the study period. Only those cases specifically identified to have RRV infection (*n* = 1,464, 93.3%) were retained for evaluation, with cases showing unspecified viral variant (*n* = 23, 1.5%) or bat variant (*n* = 82, 5.2%) excluded. Unfortunately, the database does not provide numbers of uninfected animals tested, and it was not possible to assess data accuracy independently.

Data regarding human population size by county were obtained from the US Census bureau using data from the 2000 and 2010 Censuses, in addition to the intercensal estimates for intervening years ( US Census Bureau, 2017), and human population density was calculated using county areas, as provided by the US Geologic Survey in the 2006 National Land Cover Database (US Department of the Interior, 2017). All data were compiled in Microsoft Excel spreadsheets.

### Data analyses

The statistical programming platform R (version 3.4.2) was used to evaluate the data, employing the glmmADMB package (version 0.8.3.3) (Skaug et al., 2016; R Core Team , 2017). This package was chosen due to its ability to handle a wide variety of modelling approaches, thereby maintaining consistency of analysis. Given that the data were count data, Poisson and negative binomial distributions were considered for possible use. During exploratory analysis, the overall mean annual number of cases was found to be 1.66, with a variance of 13.94. This indicated that the data were over-dispersed and that negative binomial modelling was most appropriate. However, negative binomial modelling requires an offset variable to denote the population at risk in each cluster during regression, and to reflect different weighting of the data clusters. Although the preferred offset would be total raccoon population in each county, these data are not readily available. Several potential candidate offsets were evaluated in exploratory analyses, including area (in km^2^) of counties, total county human population and county human population density. It became evident that these potential offsets were essentially equivalent, both in coefficient value as well as *p*-value. Given the known behavior of raccoons and their propensity to inhabit areas in close proximity to human activity, human population density in each county was selected as the offset, as it was believed to be most likely to be proportional to actual raccoon populations (Erb et al., 2012). In addition, human population density is also relevant to exposure risk, insofar as the majority of animals submitted came through the public health surveillance system following human or domestic animal exposures.

**Figure 1:**
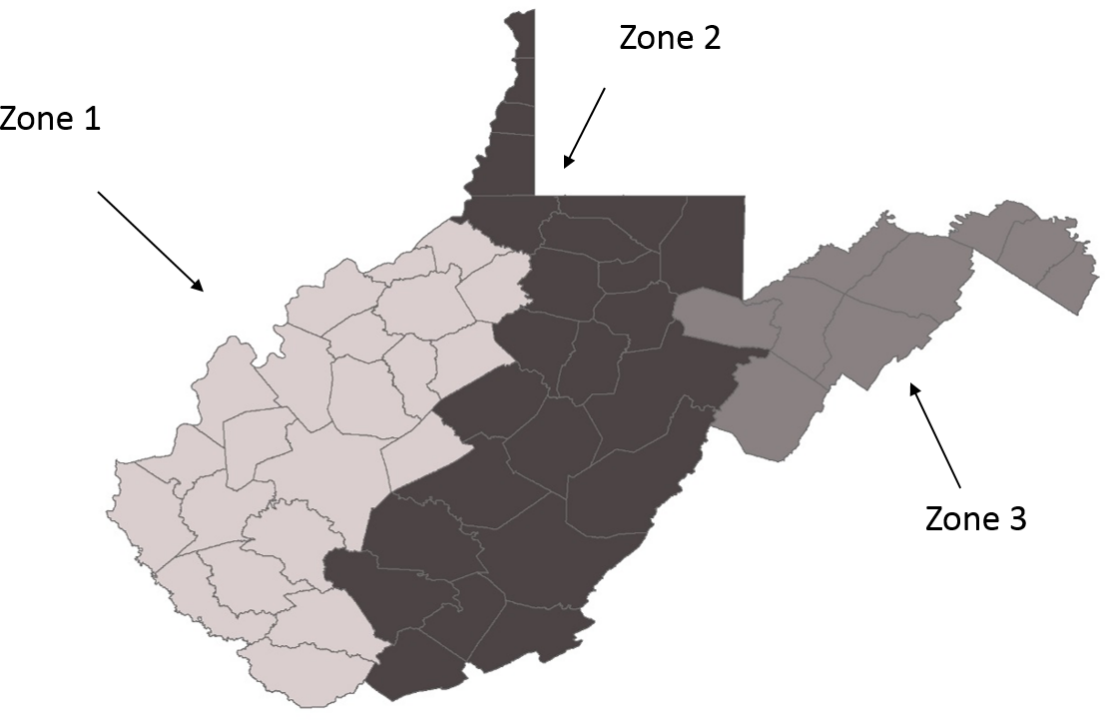
Map of the zones in West Virginia, as designated for this study. A map of the three zones for the state of West Virginia as determined by raccoon variant rabies virus (RRV) presence and oral rabies vaccine (ORV) distribution (Zone 1, No RRV during study period; Zone 2, RRV present and ORV distributed; Zone 3, RRV present, ORV not distributed).

Several counties in the western portion of WV had no positive samples for RRV throughout the study period, as a result of the failure of RRV to reach these western areas. It was decided to restrict the analysis to those counties that had at least one positive sample in the period 2000–2015 (Table 1). The state was divided into three zones. Zone 1 included all counties that experienced no RRV positives during the study period and thus were excluded from the analysis. Zone 2 comprised those counties that reported a RRV positive animal and where ORV baits were distributed for the years 2005–2015 (R Chipman, pers. comm., 2017; United States Department of Agriculture, 2014), and Zone 3 was all remaining counties where RRV was reported but ORV was not deployed (see Table 1 and Fig. 1). Analyses were run for all host species combined, as well as for raccoons, nondomestic non-raccoons (NDNR) and domestic animals separately. The NDNR grouping includes all non-raccoon wildlife. Additionally, the combined zones, as well as each zone separately, were analyzed for each of these animal groups.

**Table 1:** Counties contained within each of the zones, West Virginia. Each county within West Virginia was assigned to a zone based on the presence/absence of RRV as well as whether ORV baits were distributed during the study period, 2000–2015.

**Zone 1** Counties that reported no RRV cases in the study period (*n* = 22)	Boone, Cabell, Calhoun, Clay, Doddridge, Gilmer, Jackson, Kanawha, Lincoln, Logan, Mason, McDowell, Mingo, Pleasants, Putnam, Ritchie, Roane, Tyler, Wayne, Wirt, Wood, Wyoming
**Zone 2** Counties that reported at least 1 RRV case and had ORV baits distributed during the study period (*n* = 24)	Barbour, Braxton, Brooke, Fayette, Greenbrier, Hancock, Harrison, Lewis, Marion, Marshall, Mercer, Monongalia, Monroe, Nicholas, Ohio, Pocahontas, Preston, Randolph, Summers, Raleigh, Taylor, Upshur, Webster, Wetzel,
**Zone 3** Counties that reported at least 1 RRV case and did not have ORV baits distributed during the study period (*n* = 9)	Berkeley, Grant, Hampshire, Hardy, Jefferson, Mineral, Morgan, Pendleton, Tucker

A *z*-score analysis was performed to detect whether there were significant differences between the two zones for each host animal group, as well as for the combination of all animal hosts. *z*-scores, and associated *p*-values, were calculated using the standard errors and coefficient values derived from the negative binomial analyses. All models were run using *α* = 0.05 as the significance threshold.

## Results

**Figure 2:**
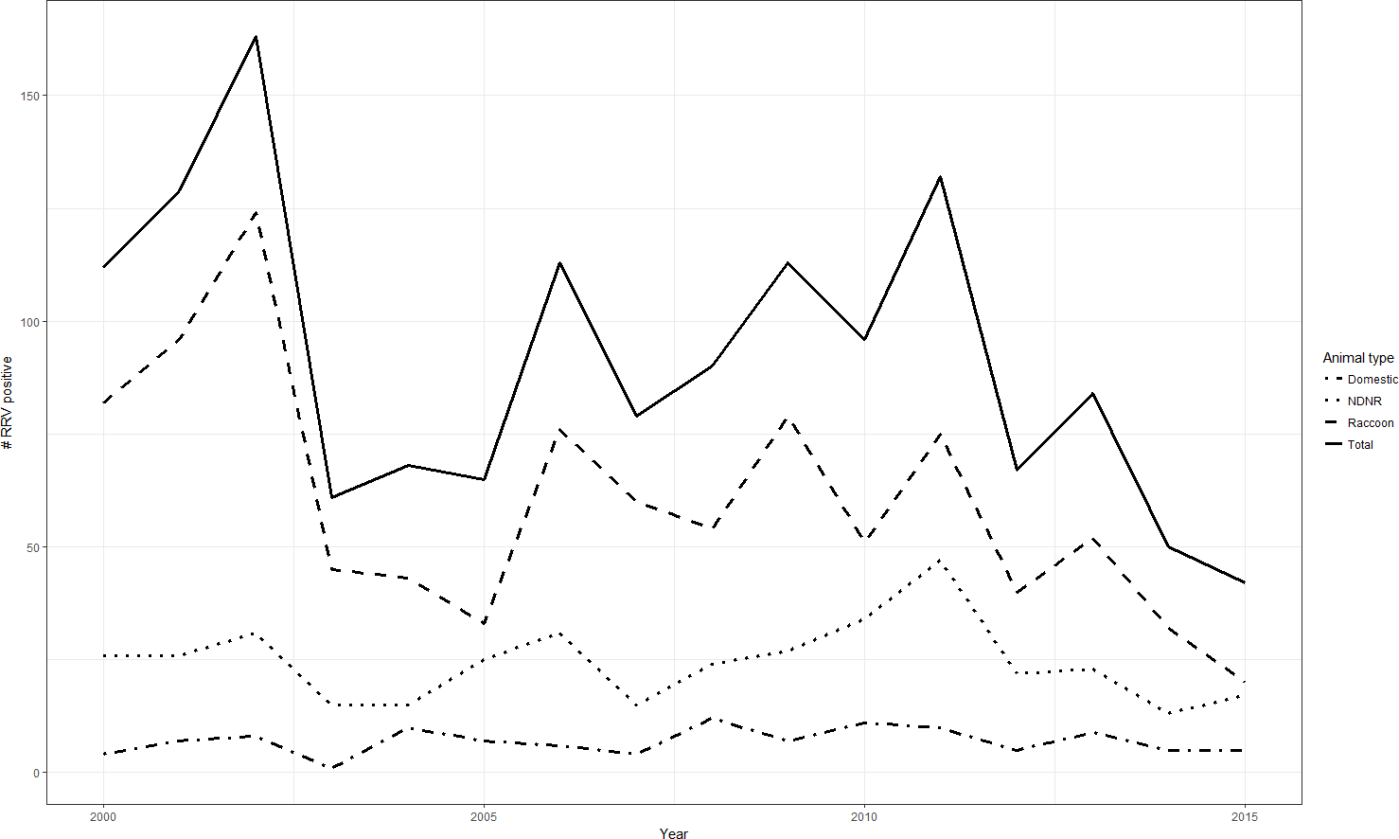
Numbers of raccoon variant rabies virus cases by animal type and year, West Virginia, 2000–2015. Graph showing trends in raccoon variant rabies virus cases for the varying animal types in this study, West Virginia, 2000–2015. Note that the overall declining trend in total cases and raccoons does not hold for NDNR and domestic animals. NDNR, Nondomestic, non-raccoons (all non-raccoon wildlife).

There were 1,464 RRV positive animals during the study period, from 2000–2015. These were comprised of 962 raccoons, 391 NDNR and 111 domestic animals. A complete species breakdown of the positive samples in non-raccoons is provided in Table 2. Preliminary examination of the state RRV data from 2000–2015 seems to show an overall decreasing trend in the number of animal RRV cases over that timeframe, as shown in Fig. 2. However, there does not appear to be a similar decline in cases for the NDNR and domestic animal hosts.

**Table 2:** Species breakdown of all non-raccoon wildlife and domestic animals infected with raccoon variant rabies virus, West Virginia, 2000–2015. Species and case count for nondomestic, non-raccoon (NDNR) animals and domestic animals diagnosed with raccoon variant rabies virus (RRV), West Virginia, 2000–2015.

	Number of RRV positive samples
NDNR species	
Striped skunk (*Mephitis mephitis*)	290
Red Fox (*Vulpes vulpes*)	70
Bobcat (*Lynx rufous*)	14
Groundhog (*Marmota monax*)	10
Beaver (*Castor canadensis*)	4
Opossum (*Didelphis virginiana*)	1
River otter (*Lontra canadensis*)	1
Bat (Species unknown)	1
Total NDNR	**391**
Domestic species	
Cat (*Felis catus*)	73
Cow (*Bos taurus*)	15
Dog (*Canis lupus familiaris*)	8
Horse (*Equus caballus*)	8
Goat (*Capra aegagrus hircus*)	4
*Sheep (Ovis aries)*	3
Total Domestic	**111**

The negative binomial model was fit for Zones 2 and 3 combined, using log human population density as the offset, yielding a regression coefficient of −0.06 with a *p*-value of <0.001 as shown in Table 3. When analyzed separately, Zone 2 had a regression coefficient of −0.09 with a *p*-value of <0.001, while Zone 3 had a regression coefficient of −0.04 with a *p*-value of <0.001. In these models the coefficients can be interpreted as follows: the mean number of cases in log-scale for Zones 2 and 3 combined was reduced by 0.06 per year for 16 years, which is equivalent to a reduction of 1.062 cases per year for 16 years. The result for Zone 2 was equivalent to a reduction of 1.094 cases per year, while the result for Zone 3 translates to a reduction of 1.041 cases per year.

**Table 3:** Comparison of negative binomial models fit for RRV in all hosts, West Virginia, 2000–2015. Negative binomial models fit for RRV in all host species. All models show significant declines in RRV cases in each zone.

Area analyzed	Coefficient (SE)	*p*-value
Zones 2 & 3 combined	−0.06 (0.011)	<0.001
Zone 2	−0.09 (0.018)	<0.001
Zone 3	−0.04 (0.012)	<0.001

**Table 4:** Comparison of negative binomial models fit for RRV in the different animal types and zones, West Virginia, 2000–2015. Negative binomial model regression coefficients and *p*-values for the various animal types in this study. Note that while raccoons showed significant declines in all models, NDNR and domestic animals did not show similar significance.

Animal type	Regression coefficient (SE)	*p*-value
Raccoon		
Zones 2 & 3 combined	−0.083 (0.013)	<0.001
Zone 2	−0.102 (0.02)	<0.001
Zone 3	−0.061 (0.014)	<0.001
NDNR^a^		
Zones 2 & 3 combined	−0.02 (0.015)	0.18
Zone 2	−0.032 (0.025)	0.21
Zone 3	−0.015 (0.018)	0.40
Domestic animals		
Zones 2 & 3 combined	0.013 (0.026)	0.62
Zone 2	−0.018 (0.04)	0.66
Zone 3	0.037 (0.035)	0.29

**Notes.**

a NDNR, Nondomestic, non-raccoon (all non-raccoon wildlife).

Negative binomial models were fit for each of the three animal types in this study, and the results are shown in Table 4. Raccoons were found to have regression coefficients of −0.083, −0.102 and −0.061, all with *p*-values of <0.001 for the zones combined, Zone 2, and Zone 3 respectively. All of these values are indicative of an increased rate of decline as compared to the results obtained for the total numbers of RRV positive animals. NDNR hosts in the zones combined had a regression coefficient of −0.02, with a *p*-value of 0.18, while zone 2 had a regression coefficient of −0.032, and a *p*-value of 0.21. Zone 3 yielded a regression coefficient of −0.015, and a *p*-value of 0.4 for NDNR. Finally, domestic animals had a regression coefficient of 0.013 and a *p*-value of 0.62 for the zones combined. Zone 2 had a coefficient of −0.018 and a *p*-value of 0.66 for domestic animals, while the same group in zone 3 had a coefficient of 0.037 and a *p*-value of 0.29. These results indicate that while there was a significant (*p* < 0.001) reduction in raccoon infection, no such significant reduction was detected in non-raccoon hosts, whether nondomestic or domestic (*p* = 0.18 and 0.62, respectively). The declines in RRV cases were statistically significant for all hosts combined, as well as raccoons, in all areas examined.

**Table 5:** Results of *z*-score analyses comparing Zone 2 with Zone 3 for all host types, West Virginia, 2000–2015. *Z*-scores indicate there is a significant difference in RRV decline for Zone 2 as compared to Zone 3 for all hosts and raccoons, with Zone 2 having a greater decline. There was not a significant difference between the zones for NDNR and domestic hosts.

Group	*z* score	*p*-value
All host species	−2.31	0.010
Raccoons	−1.67	0.047
NDNR^a^	−0.55	0.291
Domestic animals	−1.03	0.150

**Notes.**

a NDNR, Nondomestic, non-raccoons (all non-raccoon wildlife).

The results of the *z*-score analyses are presented in Table 5. When comparing the model results between Zones 2 and 3, all host species combined and raccoons were found to have significant differences (*p*-values of 0.01 and 0.047, respectively) in the rate of decline. No such significance was found for NDNR and domestic animals (*p*-values of 0.291 and 0.15, respectively).

## Discussion

There have been several studies regarding rabies virus infection, with many specific to RRV. Rabies virus is frequently reported in the eastern United States, where the primary reservoir for the virus is the raccoon (Wallace et al., 2014). Raccoons represented 32% of the positive animals nationwide in 2012, 2013 and 2014, although there was a reduction in total numbers of positive raccoons detected for these years of 1.4%, 2.8% and 4.0%, respectively (Dyer et al., 2013; Dyer et al., 2014; Monroe et al., 2016). Many prior studies regarding RRV have tended to focus on cases in raccoons and a limited number of other species (skunks, cats, rodents), without examining cases in other domestic and NDNR animals (Childs et al., 1997; Gordon et al., 2004; Guerra et al., 2003; Wallace et al., 2014). One study that did examine all species infected with RRV was primarily focused on the economic costs of rabies prevention (Gordon et al., 2005). Similarly, there have been relatively few published studies regarding longitudinal analysis of RRV that have examined all infected hosts in a specific region and time period with the intent of comparing the incidence of RRV in those hosts (Childs et al., 2001; Childs et al., 2000; Gordon et al., 2005).

Since the inadvertent introduction of RRV into the Mid-Atlantic States, the disease has spread throughout the region and into New England and Canada (Rosatte et al., 2006; Rupprecht, Hanlon & Hemachudha, 2002; Smith et al., 2005). The primary finding of the current analysis is that there has been a significant decline in all RRV positive animals in Zones 2 and 3 during the study period, with the bulk of the decline in positives occurring in raccoons in all areas examined. The rate of decline in Zone 2 is significantly greater, indicating the impact of the ORV program on RRV in that area. Substantial resources have been used in WV to control rabies virus in raccoons with apparent success (Nelson, 2010; Slate et al., 2009; Slate et al., 2014; Slate et al., 2002). However, the decline in RRV cases does not extend to non-raccoon hosts. This may reflect that the ORV bait used is less attractive to host animals other than raccoons (Slate et al., 2009). Regardless, control efforts do not appear to have significantly affected RRV infection in other hosts, as demonstrated by the results of the analysis (Table 4). Additionally, no difference between the zones was found with regard to non-raccoon hosts. The uncoupling of RRV exposure and vaccination efficacy from raccoons to other hosts suggests control efforts may enable the virus to become established in non-raccoon hosts, where it could begin to circulate independently from the raccoon reservoir. The most likely hosts that could serve as this potential reservoir would be skunks and red foxes, especially given that these hosts act as the primary host reservoir in other areas of North America. Skunks act as reservoir hosts in the central United States, while red foxes have been reservoirs historically in Canada (Dyer et al., 2013; Dyer et al., 2014; Monroe et al., 2016). This would be plausible if RRV has the postulated ability to spillover into non-raccoon hosts and potentially establish itself in new reservoir host animals (Wallace et al., 2014). However, our study did not show any evidence of their involvement as a reservoir at this time.

Our results are consistent with the results reported by Wallace et al. When we calculated cross species transmission (CST) rates as described in their paper (# non-raccoon cases / # raccoon cases), we obtained overall rates, as well as skunk and fox rates (0.52, 0.30 and 0.07 respectively), that were comparable to those found by the Wallace team in 2011 (0.73, 0.35, and 0.18) (Wallace et al., 2014). It may be encouraging that the CST rates found in our study were consistently lower than those found in 2011 by Wallace. However, our optimism is tempered by the finding that when CST rates in WV for 2011 are calculated, the overall rates, and those for skunks (0.86 and 0.54, respectively), were substantially greater than those the Wallace group reported for 2011 previously, although the CST rate for red foxes (0.08) was less than half that found by Wallace (Wallace et al., 2014).

Interestingly, when CST in 2011 is examined for each of the zones, Zone 2 had markedly higher rates than Zone 3 for all CST, as well as skunks (0.84 and 0.67 vs. 0.63 and 0.33, respectively), while CST in red foxes was substantially lower for Zone 2 (0.03 vs.0.13). While these samples are too small to make statistical inference, they are of interest nonetheless.

The findings of a reduction in RRV cases in raccoons due to ORV are consistent with the available literature. For example, Ma et al. (2010) noted a general reduction in numbers of RRV positive raccoons recovered in areas of WV where ORV occurred, subsequent to the commencement of the ORV program. Their study examined raccoons in a limited number of counties where ORV had been provided, however, and compared them to the eastern WV counties where RRV is enzootic. Here we extended their observations to include RRV induced disease in non-raccoon animals, both domestic and non-domestic, and all counties where ORV was deployed. Their data extended up through 2007 and captured 2002, the peak year of RRV positives in the state shown in Fig. 2. The current study period continues through 2015 and includes additional peaks in 2009 and 2011. The peaks described by Ma et al. (2010), and those seen in the current study, are consistent with prior descriptions of epizootic and inter-epizootic RRV, where the first epizootic was largest, with subsequent, smaller epizootics (Childs et al., 2000; Gordon et al., 2004).

There are several potential reasons for the overall reduction in RRV incidence over the study period. The ORV project is well established in the state, and our work and that of Ma et al. (2010) clearly show it is having a significant effect on overall RRV numbers. Additionally, given the rapidly fatal progression of the disease in infected animals, it is possible that it is “burning itself out” and has reached, or is reaching, a self-limiting steady state. Fluctuations in state and local human populations may also be affecting raccoon numbers, and subsequently influencing contact rates with infected animals.

The temporal pattern of RRV infection in non-raccoon animals may be cause for concern. Although prior studies have indicated that it may not be the case in cats (Gordon et al., 2004), one would anticipate that as the numbers of RRV positive raccoons decline, numbers in non-raccoon hosts would experience a similar decline. This is not borne out by the data examined here. NDNR and domestic hosts had no significant changes in RRV positive animals. This would indicate that RRV is not experiencing a decline in these animal hosts, and could be conducive to the virus becoming independently established in other reservoir(s) (e.g., skunks and foxes) where baits are not effective. The fact that neither NDNR nor domestic hosts is declining tends to decrease the likelihood that this is simply a reflection of diminished domestic animal vaccination practices. The rates of vaccination are likely to be higher in dogs than in livestock or cats (especially feral cats), and this may be reflected in the higher numbers of positives found in cats and cattle. The maintenance of steady RRV incidence is of particular concern in domestic animals, even though they are not a likely alternative host reservoir, as these are most likely to have close contact with humans. Additionally, there were a number of cases in livestock hosts (such as horses, cows, sheep and goats). Although less frequently encountered than in domestic carnivores, these cases may actually represent a greater risk to humans due to a lowered index of suspicion among farmers or veterinarians caring for these animals. Unrecognized exposures in turn could cause significant delay in proper diagnosis of these infections, potentially allowing owners and others to have greater risk of serious disease.

*Limitations:* The data available constrained the current study. Given that the data provided are restricted to county and year of collection, it is impossible to examine seasonal patterns or perform more detailed geographic analysis. Additionally, it is difficult to assess whether the animals submitted for testing are truly representative of the disease as it exists in the larger natural population of these hosts within the state. It seems likely that these animals represent a biased sample of the population as a whole. This bias could easily result in an underestimation of the true impact of RRV. Finally, the limited number of submissions of non-raccoon animals, both domestic and wild, may place constraints on accurate assessment of the longitudinal trend in these hosts.

Another significant limitation of the study is the lack of data regarding the species and total number of all animals tested. As a result, it is difficult to assess whether surveillance efforts remained consistent throughout the study period. However, it seems likely that surveillance efforts would have remained constant, or may have increased, due to the implementation of the ORV program, to determine its efficacy and to justify continued funding. This potential elevation of surveillance would be unlikely to extend to host species other than raccoons, nor would it likely extend beyond the counties where the ORV program was implemented and would be unlikely to have resulted in the reduction in RRV cases found in this study.

## Conclusion

This study demonstrates that numbers of RRV positive animals declined significantly over the study period in those areas of the state of WV where RRV is enzootic, particularly in the primary viral reservoir host, raccoons. There is no reason to assume that diagnostic or recovery methods have changed during this same period. The results of this study would indicate that public health efforts are needed to improve vaccination rates in all domestic animals, including livestock, especially in those counties where RRV is enzootic. Public information campaigns targeted at veterinarians and livestock owners could be instituted in those areas to increase awareness of the risk of rabies infection. Further examination of RRV in non-raccoon hosts seems warranted to explain why these diverse groups are not trending down along with the raccoons. Future directions for this work include a spatial analysis of those factors that may be associated with RRV and raccoon populations, including land use, human population density and availability of surface water as well as ambient weather conditions. Additionally, cluster analysis of RRV positives would provide useful information to use as guidance for RRV control and other public health measures.

##  Supplemental Information

10.7717/peerj.4574/supp-1 Supplemental Information 1 Raw data as used for the study

10.7717/peerj.4574/supp-2 Supplemental Information 2 Melt file for use in preparing Fig. 2

10.7717/peerj.4574/supp-3 Supplemental Information 3 R code used for data analysis

## Additional Information and Declarations

### Data Availability

The following information was supplied regarding data availability: The raw data are provided in the Supplemental Files.
